# Peer-Influence on Risk-Taking in Male Adolescents with Mild to Borderline Intellectual Disabilities and/or Behavior Disorders

**DOI:** 10.1007/s10802-018-0448-0

**Published:** 2018-06-26

**Authors:** Anika Bexkens, Hilde M. Huizenga, David A. Neville, Annematt L. Collot d’Escury-Koenigs, Joren C. Bredman, Eline Wagemaker, Maurits W. Van der Molen

**Affiliations:** 1 Department of Psychology, University of Amsterdam, Amsterdam, Netherlands; 2 Department of Psychology, Developmental and Educational Psychology, Leiden University, Wassenaarseweg 52, PO 9555, 2300 RB Leiden, The Netherlands; 3 Heeren Loo Groot Emaus, Groene Allee 46, 3853 JW Ermelo, Netherlands; 4 Amsterdam Brain and Cognition, University of Amsterdam, Amsterdam, The Netherlands; 5 Research Priority Area Yield, University of Amsterdam, Amsterdam, The Netherlands; 6 Donders Institute for Cognitive Neuroscience, Radboud University, Nijmegen, The Netherlands

**Keywords:** Adolescent risk-taking, Attention deficit hyperactivity disorder, Disruptive behavior disorder, Intellectual disability, Peer influence, Balloon analogue risk-task

## Abstract

This study aimed to disentangle the effects of Mild-to-Borderline Intellectual Disability (MBID) and Behavior Disorders (BD)on risk taking in circumstances where peer influence was absent or present. We studied 319 adolescents in four groups: MBID-only, MBID+BD, BD-only, and typically developing controls. The Balloon Analogue Risk-Task (BART), in a solo or peer condition, was used as a proxy of real-life risk-taking. Results show a significant main effect of BART condition. Post-hoc tests indicated higher risk-taking in the peer compared to the solo condition in all groups except BD-only. Moreover, risk taking was increased in adolescents with MBID compared to adolescents without MBID, but only under peer-influence. No main or interaction effects with BD were observed. Model based decomposition of BART performance in underlying processes showed that the MBID related increase in risk-taking under peer-influence was mainly related to increased risk-taking propensity, and in the MBID-only group also to increased safety estimates and increased confidence in these safety estimates. The present study shows that risk-taking in MBID may be better explained by low intellectual functioning than by comorbid BD, and may not originate in increased risk taking per se, but may rather be related to risk-taking under peer-influence, which is a complex, multifaceted risk-taking context. Therefore, interventions to decrease risk-taking by adolescents with MBID that specifically target peer-influence may be successful.

Adolescence is a period of great strength and resilience during which both physical and mental capabilities increase rapidly. However, it is also a period of high risk for negative outcomes (Forbes and Dahl 2010). Mortality and morbidity rates double during this period, not because of a steep rise in illnesses, but rather due to an increase in risk-taking behavior such as substance abuse, unsafe sex, and reckless driving (Institute of Medicine 2011). The dominant explanation in the literature for adolescent risk-taking is related to an imbalance in brain development with a protracted development of the cognitive control system relative to high reactivity in the reward system (Steinberg 2010). Although risk-taking behavior seems common in adolescence, there are in fact many individual differences (see Jessor 1991), which have been related to individual differences in sensation-seeking (Duell et al. 2016; Harden and Tucker-Drob 2011), impulsivity (Harden and Tucker-Drob 2011; Vermeersch et al. 2013) and cognitive control (Pharo et al. 2011; Vermeersch et al. 2013).

Real-life risk-taking typically occurs when peers are present (Albert and Steinberg 2011). Not only do adolescents spend an increasing amount of time in presence of peers (Larson et al. 1996; Steinberg and Morris 2001), they also become increasingly sensitive to the influence of peers (Crone and Dahl 2012; Duell et al. 2016; Van Hoorn et al. 2016a). Laboratory studies indicate that adolescents show enhanced risk taking in presence of peers, whereas adults do not (Chein et al. 2011; Gardner and Steinberg 2005). Moreover, during adolescence structures responsive to reward are also sensitive to social stimuli (Steinberg 2010). Therefore, individual differences in adolescent risk-taking cannot and should not be studied without considering vulnerability to peer-influence. Chein et al. (2011) show that peer presence enhances *reactivity in* the brain’s reward circuitry, thereby increasing risk-taking, suggesting an influence of presence of peers on reward valence.

However, literature on the adolescent social brain points to another pathway between peer-influence and increased risk-taking that is related to social-cognitive processes. There is evidence that the relationship between lower resistance to peer-influence and increased behavioral risk-taking is mediated by right temporoparietal junction (TPJ) response (Falk et al. 2014; Peake et al. 2013). TPJ is considered to be one of the core regions associated to social-cognitive processes such as perspective-taking (Carrington and Bailey 2009), suggesting an important role for social-cognitive processes during peer-influence on risk-taking. In sum, factors related to the explanation of adolescent risk-taking are cognitive control processes, reactivity of the reward system to reward and social cues and social-cognition.

The focus of the present study is on risk-taking in adolescents with Mild-to-Borderline intellectual disability. Studies specifically targeting risk-taking in this group show increased real-life risk taking with respect to substance use, sexual risk-taking and delinquency (Chapman and Wu 2012; Emerson and Halpin 2013; Holland et al. 2002; McGillivray 1999). In the current study, Mild-to Borderline Intellectual Disability is defined by borderline intellectual functioning (IQ between 70 and 85) or mild intellectual disability (IQ between 50 and 85) in combination with significant limitations in adaptive functioning as described in the DSM-IV (DSM-IV- American Psychiatry Association 2000; Schalock et al. 2010), which was current when the study was conducted. With the introduction of DSM-5, the severity of the intellectual disability is no longer defined by IQ, but by severity of limitations in adaptive functioning. Prevalence of MBID is about 10% (Simonoff et al. 2006) and it is a heterogeneous group with about 40% of children and adolescents with MBID also meeting criteria for mental health disorder, such as anxiety, mood, or disruptive behavior disorder (as is reported in Dekker and Koot 2003).

With regard to risk-taking, low intellectual functioning is a well-known risk factor for adolescent real-life risk-taking, such as rule-breaking and delinquent behavior. From the perspective of the imbalance model of adolescent risk-taking, increased risk-taking in MBID could be explained by a potential larger imbalance between cognitive control and reward sensitivity in this group. Due to a lack of research in this area it is not clear whether adolescents with MBID would be more sensitive to rewards than adolescents without MBID. There is ample evidence, however, that MBID is associated to cognitive control deficits (for a meta-analysis see Bexkens et al. 2014a).

With regard to increased susceptibility to peer-influence, this is something that is often reported by professionals working with this population, but which has been understudied. There is some evidence from vignette studies that adolescents with MBID struggle to make safe decisions under negative peer-pressure (Khemka et al. 2009; Khemka et al. 2016). However, these studies did not include a comparison to typically developing adolescents and therefore could not provide evidence of a higher susceptibility to peer-influence in the MBID group than in typically developing adolescents. From the perspective of the imbalance model, increased susceptibility to peer-influence could be explained by a higher limbic reactivity to social cues. There has been no research that targeted this question. There is however evidence of social cognitive deficits (Abbeduto et al. 2004). These deficits would make adolescents with MBID potentially more vulnerable to peer-pressure because they would be less able to read their peers’ intentions (Abbeduto et al. 2004; Greenspan et al. 2011; Khemka et al. 2009).

As stated above, MBID is a heterogeneous group. Although it would be possible to just focus on a relatively homogeneous group of adolescents with MBID without comorbid problem behavior, this would result in low generalizability of results to clinical practice. Many adolescents referred for risk-related behavior also present with comorbid problems, especially externalizing problems. On the other hand, from an experimental perspective, it would be better to study a homogeneous population to limit alternative explanations and increase power. In order to attain both of these goals (i.e., relevant to real-life clinical practice and limit alternative explanations and sufficient power) we included treatment resistant behavioral problems (BD) as a factor in the study. This type of behavior problems lead to a diagnosis of Conduct Disorder, Oppositional Defiant Disorder and Attention Deficit Hyperactivity Disorder (American Psychiatry Association 2000) in 25–30% of children and adolescents with MBID (Dekker and Koot 2003; Emerson and Hatton 2007). Thus, prevalence of childhood behavioral disorder (BD) is quite high in MBID considering a prevalence of 5–10% in the general population. BD’s are of specific interest to the present study as BD is also associated to enhanced risk taking in both real life (Barkley et al. 1993; Elkins et al. 2007; Jessor 1991; Ramrakha et al. 2007) and experimental contexts (Dekkers et al. 2016; Schutter et al. 2011). BD has also been linked to aberrant reward processing and cognitive control problems (Alegria et al. 2016) which may explain increased risk-taking in this group. With respect to peer-influence, there is substantial literature about the role of peer-influence in the *development* of externalizing problem behavior showing the role of peer-modelling and positive reinforcement of deviant behavior (Dishion and Tipsord 2011). It is however not clear whether adolescents with BD are also more susceptible to peer-influence than typically developing adolescents.

In the current study, risk-taking was assessed using the Balloon Analogue Risk Task (BART) in conditions without or with peer influence. The BART, which is a computerized task, has been proven to be a particularly useful tool to examine risk-taking (Lejuez et al. 2002; Wallsten et al. 2005). The BART is a computerized task measuring risk-taking propensity. Participants performing the BART are presented with an empty balloon to inflate, receiving a small amount of money for each pump. At any time, the individual has the opportunity to stop pumping and collect the money accrued or to continue pumping while running the risk that the balloon pops losing the accumulated money. Pumping is risky business, as the probability that a balloon will pop increases with each pump. It is rewarding at the same time since each additional pump will increase the amount of money gained. Its construct validity was supported by significant relations to a range of daily life risk-taking behaviors (Hunt et al. 2005; Lejuez et al. 2007, 2002; MacPherson et al. 2010; Mishra et al. 2010; Wallsten et al. 2005). In addition, White et al. (2008) indicate a test-retest correlation of 0.77 between test days, indicating that task performance on the test day is likely to be representative of task performance on another day. Traditionally, BART performance is analyzed by focusing on the average number of times participants pumped up the balloons that did not explode (i.e., adjusted pumps). We went beyond the traditional analysis of BART performance by applying a cognitive processing model of risk taking behavior in the BART developed in previous studies (Cavanagh et al. 2012; Hunt et al. 2005; Van Ravenzwaaij et al. 2011; Wallsten et al. 2005). This model was used to decompose risk-taking behavior into latent factors and allowed for a more in depth analysis of group differences and peer-influence effects on risk-taking behavior.

The peer manipulation used in the present study was modelled on previous research on peer-influence on adolescent risk-taking (Gardner and Steinberg 2005). Three characteristics are important. First, in contrast to the paradigm used by Gardner and Steinberg (2005) we standardized the type of peer-encouragement each adolescent received by using virtual unknown peers. The use of unknown peers was relevant as it prevented variation in peer-relationship dynamics, which could potentially differ between adolescents from different clinical groups. Second, similar to Gardner & Steinberg, we allowed for active peer encouragement. Although recent studies indicate that the presence of peers is sufficient to affect behavior (Chein et al. 2011; Smith et al. 2014), several studies that compared peer presence and active peer encouragement indicate stronger effects in peer encouragement conditions (Falk et al. 2014; Reynolds et al. 2014; MacLean et al. 2014). Third, the content of the risk-encouraging statements was mixed in that it combined reinforcement of taking risk (‘risk is cool’) with discouragement of safe decisions (‘stopping is lame’). This type of statements is ecologically valid. These encouragements were thus explicit, as there is evidence from observational studies that deviant peer-influence works in part through explicit reinforcement (Dishion and Tipsord 2011). However, note that peer-influence can also be very subtle, for instance it may also work via imitation (Larsen et al. 2012).

To disentangle the effects of MBID, BD and peer-influence on risk-taking behavior we adopted a 2 MBID (present vs absent) by 2 BD (present vs absent) by 2 BART condition (solo vs peer) between subjects design. This resulted in the inclusion of four adolescent populations: typical control, BD-only, MBID-only and MBID+BD.

We hypothesized that both MBID and BD would be associated with increased risk-taking behavior as MBID is associated to cognitive control problems (Bexkens et al. 2014a) and BD to both increased reward sensitivity and cognitive control problems (Alegria et al. 2016). In addition, we expected increased susceptibility to peer-influence for both BD and MBID. As discussed above, evidence from clinical observation (e.g., Greenspan et al. 2011) and vignette studies (Khemka et al. 2009) indicate susceptibility to peer-influence in MBID. In addition, the imbalance model would predict an increase in limbic activity in response to peers that adolescents with MBID and adolescents with BD would be less able to downregulate due to impaired cognitive control (Alegria et al. 2016; Bexkens et al. 2014a).

## Methods

### Participants

A total of 352 male adolescents between 12 and 18 years of age (*M*_age_ = 14.9, *sd* = 1.5) participated in the study. Inclusion criteria were intelligence level (above or below 85) and presence or absence of DSM-IV diagnoses of childhood behavior disorders (ADHD, ODD, or CD). Based on these criteria, each adolescent was assigned to one of four groups: typical controls, BD-only, MBID-only, and MBID+BD. Descriptive statistics of the four groups of participants are presented In Table 1. Adolescents were recruited from different school types that matched the study inclusion criteria. Participants in the control group were recruited from regular education schools. Participants in the other groups were recruited from special education schools. School types matched the three clinical groups (BD, MBID, or MBID+BD) and use strict admittance criteria (see below) based on the specific school type. In addition, we used information on IQ and DSM-IV diagnosis from school files to refine this selection where needed. The same procedure has been described in Bexkens et al. (2014b). Descriptive statistics of the four groups of participants are presented In Table 1.

**Table 1:** Participant characteristics for each of the four groups

	Control (*N* = 92)	BD-only (*N* = 75)	MBID-only (*N* = 76)	BD + MBID (N = 76)
Mean age	14.6 (1.4)_a_	14.7 (1.3)_a_	15.2 (1.7)_b_	15.1 (1.6)_a_
Mean IQ	–	96.6 (8.3_)a_	71.4b (11.8)_b_	71.4 (9.6)_b_
SPM-IQ	95.8 (11.8)_a_	92.6 (10.5)_a_	72.8 (13.8)_b_	72.4 (13.2)_b_
DSM-IV disorders		18 ADHD 38 DBD 19 ADHD+DBD		17 ADHD 41 DBD 18 ADHD+DBD

Standard deviations are reported between parentheses. *BD*, Behavior Disorder; *MBID*, Mild-to Borderline Intellectual Disability; *ADHD*, Attention deficit/Hyperactivity Disorder; *DBD*, Disruptive Behavior Disorders, which includes Oppositional Defiant Disorder and Conduct Disorder. Reported values are calculated after exclusion of 30 participants (cf. section “Data Analysis”). Different subscript letters denote significant difference between groups, *p* < 0.05

Participants in the control group were selected from regular education schools. We used a two-step approach to assign participants to MBID and/or BD groups, which has been previously described by Bexkens et al. (2014b). We first selected participants from special education schools that corresponded to the target groups (i.e., MBID-only, BD-only and MBID+BD). These schools have the strict admittance criteria, which at the time of testing were re-evaluated once every 2 years by an independent committee.

Schools for adolescents with childhood behavioral disorders (BD group): (1) the student is diagnosed with a DSM-IV (or ICD-10) psychiatric, behavioral, or social-emotional disorder by a psychiatrist or clinical psychologist no more than 2 years prior to admittance; (2) the student displays problematic behavior across contexts (e.g., at school and home); (3) student and family support provided by child and adolescent mental health services have not led to improvement of the problem behavior; (4) the student experiences a learning impediment resulting from the diagnosed disorder such as problems with concentration or motivation, or has learning delays in at least two areas. These delays do not result from lower cognitive ability; and (5) the student’s previous school has undertaken efforts to adapt the school environment to the child’s needs, but this has not led to sufficient improvement in the student’s behavior. We only selected adolescents form this school that had a DSM-IV diagnosis of ADHD,

Schools for children with childhood behavioral disorders and MBID (BD + MBID group): Admittance criteria were the same as those for BD, with the additional criterion of an IQ between 55 and 85, tested with a standardized IQ test not more than 2 years prior to admittance.

Schools for children with MBID (MBID-only group): (1) an IQ between 55 and 85 tested no more than 2 years prior to admittance on a standardized IQ test; and (2) learning delays of 50% or more in at least two of the following areas: mathematics, reading accuracy and fluency, reading comprehension, and spelling. One of these delays must be in mathematics or reading comprehension.

These group allocations were subsequently checked by checking school files on IQ scores from standardized IQ tests (i.e., above or below 85) and DSM-IV classifications of severe, treatment resistant childhood behavior problems (i.e., Oppositional Deviant Disorder (ODD), Conduct Disorder (CD), Disruptive Behavior Disorder NOS, or Attention Deficit/Hyperactivity Disorder (ADHD)). Children with other DSM-IV classifications were not included in the study. As all adolescents in the two BD-groups where placed at schools for adolescents with externalizing behavior problems, even the children with only a diagnosis of ADHD had additional behavior problems, although these were not always diagnosed as comorbid ODD, CD or behavior disorder NOS’**.** This led to 5 reclassifications based on IQ, from the BD-only to the MBID+BD group as these participants had a recently tested IQ below 85. There were no reclassifications based on DSM-IV diagnosis of childhood behavioral problems. An ANOVA on SPM IQ-scores showed differences in the expected direction. Raven SPM may not be sensitive to all intellectual problems as it taps fluid, but not crystallized intelligence. However, all children from regular education schools had successfully finished regular primary education. As an additional precaution, teachers from regular education schools were asked to identify children whom they suspected might experience intellectual or behavioral problems, but none of the participating adolescents were identified as such. In addition, adolescents in the clinical groups had all participated in standardized IQ testing as part of admittance to special education. Comparison of the clinical groups on these standardized IQ scores from the school files showed differences in the expected direction. See Table 1 for means and standard deviations for SPM-IQ and IQ-scores from school files.

Medication use was also recorded and indicated that 19 adolescents in the BD-only group (25.3%) and 18 adolescents in the BD + MBID group (23.7%) used methylphenidate or risperidone for their behavioral problems.

Informed consent was obtained from both parents (or caretakers) and adolescents. Parents were individually contacted through the schools and asked for their consent two weeks before testing, adolescents were asked for their consent immediately before testing. It was explained to both parents and adolescents that they could withdraw from participation whenever they wanted and without any consequences. The study was approved by the ethical review board of the University of Amsterdam and complied with relevant laws and guidelines.

### Materials

#### Balloon Analogue Risk Task

**Fig. 1:**
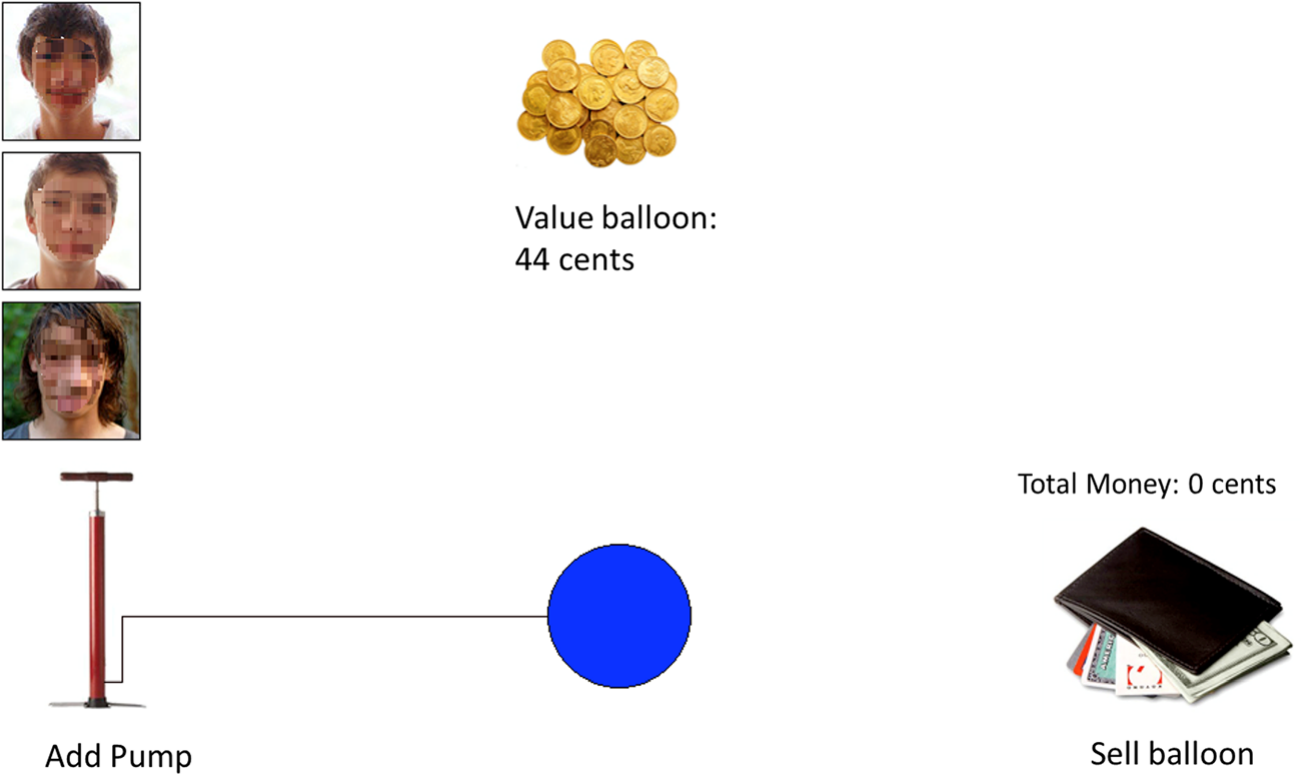
Sample trial from the peer-influence condition. Participants inflated the balloon by clicking the mouse on the pump. Each click inflated the balloon a little and was rewarded by one cent. The counter above the balloon kept track of the cents earned on a particular trial. When the balloon exploded, an explosion cartoon was presented on the screen together with an explosion sound. All cents earned on that trial were then lost and the counter above the balloon was reset to zero. Participants were instructed that they could decide to ‘sell’ the balloon at a point of their own choosing by clicking on the wallet. All points earned on that trial were then transferred to the counter above the wallet, which was accompanied by the sound of a slot machine. Three pictures of same-sex peers were displayed during each trial in the peer condition only. Audio files with risk-encouraging statements were played at random moments during the task. When an audio file played, a speech balloon appeared next to one of the pictures indicating which peer was speaking

An adaptation of the BART (Lejuez et al. 2002) was used to assess risk-taking performance. Participants were sitting behind the screen of a HP 550, 15.4 in. notebook. On each trial, participants were instructed to earn money by pumping up a balloon, whilst running the risk of the balloon popping and losing the money (cf. Fig. 1 for a picture of a BART trial).

The task consisted of 30 trials, that is, 30 balloons. We deviated from the procedure described by Lejuez et al. (2002) in that the balloon could not explode on the first 5 pumps. This way none of the balloons would pop immediately, which we expected to have a large impact on participant behavior on the task. The probability of explosion on the sixth pump was 1/123, the probability of explosion on the seventh pump was 1/122, etc. This procedure was used once to select an explosion point for every balloon. This array of explosion points was then used for all participants, so there was no inter-participant variation in the probability of balloons exploding. Although the risk of explosion increases with every pump, this was not told explicitly to the participants. The adjusted pumps, the average number of pumps on non-explosion trials, was used as dependent variable. Participants inflated the balloon by clicking the mouse on the pump (cf. Fig. 1). Each click inflated the balloon a little and was rewarded by one cent. The counter above the balloon kept track of the cents earned on a particular trial. Participants were running the risk that the balloon exploded at the next click. When the balloon exploded, an explosion cartoon was presented on the screen together with an explosion sound. All cents earned on that trial were then lost and the counter above the balloon was reset to zero. Participants were instructed that they could decide to ‘sell’ the balloon at a point of their own choosing by clicking on the picture of the wallet. All points earned on that trial were then transferred to the counter above the wallet, which was accompanied by the sound of a slot machine. (Lejuez et al. 2003, 2002).

#### Peer Influence Manipulation

Participants were assigned to the conditions randomly by alternately assigning adolescents to each condition. Random assignment was constrained by group, so that half of participants in each group was assigned to the solo and the other half to the peer condition. In the ‘solo’ condition adolescents performed the BART as described above (Solo BART). In the ‘peer’ condition (Peer BART), three pictures of adolescent boys were presented in the left-hand corner of the screen. In addition, audio files with statements encouraging risk taking were played at random moments during inflating the balloon (e.g., ‘If you stop now you are chicken’ and ‘you are really cool if you go on pumping’). Participants were told that the audio files contained advice from adolescents whose pictures were presented on screen and who had already performed the task and would make comments based on their task experience.

### Testing Procedure

All participants were tested, individually, in an empty classroom at their school. Half of the participants performed the Solo BART and half of the participants took the Peer BART. The BART has not previously been used in the MBID population. For that reason, the task was piloted in a small sample of adolescents with MBID that did not participate in the current study. Task instructions were adapted after this pilot. To standardize instructions a step-by-step instruction was added to the task with two example items. During this step by step protocol, that was programmed into the task, adolescents received instruction on the different elements on screen, how to pump up the balloon, how to cash the balloon and what would happen when the balloon exploded. After the practice trials, participants were asked to choose one of three age appropriate potential prizes worth 75 euros by clicking the corresponding picture on the screen. Participants were told they would enter a raffle and would receive one raffle ticket for each 100 BART cents. Participants took about 20 min to finish the task. All adolescents were able to perform the task. Additional instruction was provided when needed during the practice trials. All adolescents finished the task and there were no adolescents who either cashed all balloons without pumping or let all balloons explode without cashing suggesting the participants understood how to execute the task.

### The BART Model

In order to decompose BART performance into latent factors of risk taking, we used a hybrid hierarchical BART model (Cavanagh et al. 2012), based on the model developed by Wallsten and colleagues (see Wallsten et al. 2005, model 3). The model includes four parameters that describe between subject variability in risk taking (Cavanagh et al. 2012; Lejuez et al. 2003; Rolison et al. 2012; Wallsten et al. 2005).

The key risk taking parameter in the model is the first parameter, γ^+^, which indexes risk-taking propensity, or how risk-seeking one is (cf. Table 2). The γ^+^, parameter is positively related to real life risk-taking such as drug use, unprotected sex and stealing (Cavanagh et al. 2012; Rolison et al. 2012; Wallsten et al. 2005). The second parameter, β, indexes participants’ consistency in adopting their response strategy. The third and fourth parameters, *α*_*0*_ and *μ*_*0*_, provide information on perceptions of safety prior to performing the task. Using these parameters, two variables can be calculated that provide information on safety-perception: the mean and variance of the prior distribution of expected non-explosion points (Cavanagh et al. 2012). The mean of the prior distribution of estimated probabilities of non-explosion (Q_mean0_) provides an index of safety estimation, the variance of the prior distribution (Q_variance0_) indicates how uncertain the decision-maker is about this estimate, with higher number indicating higher uncertainty. As the safety-estimation and uncertainty parameters have a relatively small range, Q_mean0_ values were multiplied by 100 and Q_variance0_ values were multiplied by 1000 to allow for representation in tables and graphs.

**Table 2:** Model parameters and derived measures and their interpretations

Parameter/Measure	Interpretation	
γ^+^	Risk-taking propensity	Higher values signify higher risk-taking propensity
*β*	Behavioral consistency	Higher values signify higher behavioral consistency
Q_mean0_	Safety estimates	Mean estimated probability of non-explosion
Q_variance0_	Uncertainty in safety estimates	Variance estimated probability of non-explosion

### Data Analysis

The model fits were first checked for imprecise fits. Convergence values (Rhat) larger than 1.5^1^ were taken as indicative of imprecise fits (Cowles and Carlin 1996). Based on this procedure 20 participants (5 control, 4 BD-only, 5 MBID-only and 6 MBID+BD) were excluded. In addition, all dependent variables (adjusted pumps, risk-taking propensity, behavioral consistency, safety-estimation and uncertainty) were checked for outliers. Participants with scores that deviated three standard deviations or more from their group mean were excluded. This procedure led to the additional exclusion of 10 participants (4 controls, 3 MBID and 3 MBID+BD). Excluded participants were equally distributed over the two levels of BART condition (*χ*^*2*^ = 0.00, *p* = 0.98), the two levels of MBID (*χ*^*2*^ = 0.89, *p* = 0.35) and the two levels of BD (*χ*^*2*^ = 0.18, *p* = 0.68) and did not differ from included participants in Age, *t*(347) = −0.099, *p* = 0.98. Excluded non-MBID participants did not differ from their included counterparts in IQ, *t*(171) = 0.85, *p* = 0.40. Excluded MBID participants had somewhat lower IQ than their included counterparts, *t*(166) = 2.19, *p* = 0.03 (mean IQ’s of 65.1 and 72.6 respectively).

All dependent variables were analyzed using ANOVAs with MBID (Present vs Absent), BD (Present vs Absent) and BART condition (Peer vs Solo), as between factors. For follow-up tests, we used the Bonferroni corrected post-hoc tests in SPSS when relevant. With this option, SPSS returns *p*-values that are Bonferroni corrected by multiplying p-values by the number of tests (IBM support 2016). To be consistent, we extended this procedure also when testing simple effects. That is, to follow-up on the MBID by BART condition interactions, we tested the effect of MBID in the Peer and Solo condition separately and subsequently multiplied *p*-values by 2. To follow-up on the MBID by BD interactions, we tested all 6 contrasts between the typical controls, BD-only, MBID-only and MBID+BD groups and multiplied p-values by 6. Bonferroni corrected p-values are denoted by the addition of superscript B (*p*^B^). Additional analyses revealed that controlling for age or medication did not alter the pattern of results reported below. Moreover, within the BD groups there were no significant differences between DSM-IV diagnosis subgroups (i.e., ADHD, DBD or ADHD+DBD) on any of the dependent BART variables.

## Results

### BART Risk-Taking

#### Adjusted Pumps

To investigate effects of MBID, BD and Peer-influence on BART risk-taking, the first set of analyses was performed on the adjusted average pumps (cf. Table 3 for means and standard deviations). The ANOVA yielded significant main effects of MBID, *F*(1, 311) = 8.70, *p* = 0.003, *partial η*^*2*^ = 0.03, and BART condition, *F*(1,311) = 51,17, *p* < 0.001, *partial η*^*2*^ = 0.14.^2^ The main effect of BD was not significant, *p* = 0.62.

**Table 3:** Means and standard deviations of the variables derived from BART performance

		Typical control		BD-only		MBID-only		MBID+BD	
		*M*	*SD*	*M*	*SD*	*M*	*SD*	*M*	*SD*
Adjusted pumps	Solo	29.56	9.81	29.88	9.81	28.96	8.36	30.74	11.16
	Peer	35.13	9.65	32.95	9.48	42.55	9.80	39.44	10.87
Risk-taking propensity	Solo	0.69	0.31	0.75	0.32	0.74	0.25	0.77	0.26
	Peer	0.90	0.33	0.94	0.42	1.16	0.38	1.18	0.51
Behavioral consistency	Solo	0.10	0.04	0.10	0.05	0.08	0.03	0.09	0.03
	Peer	0.09	0.03	0.09	0.04	0.07	0.02	0.07	0.03
Safety estimate	Solo	97.05	1.14	97.02	1.33	97.02	1.60	96.41	1.65
	Peer	96.68	1.08	96.80	1.59	97.62	1.56	96.80	1.59
Uncertainty	Solo	0.75	0.27	0.76	0.31	0.75	0.38	0.90	0.39
	Peer	0.88	0.26	0.88	0.40	0.65	0.37	0.82	0.37

*MBID*, Mild-to-Borderline Intellectual disability; *BD*, Behavior Disorder

**Fig. 2:**
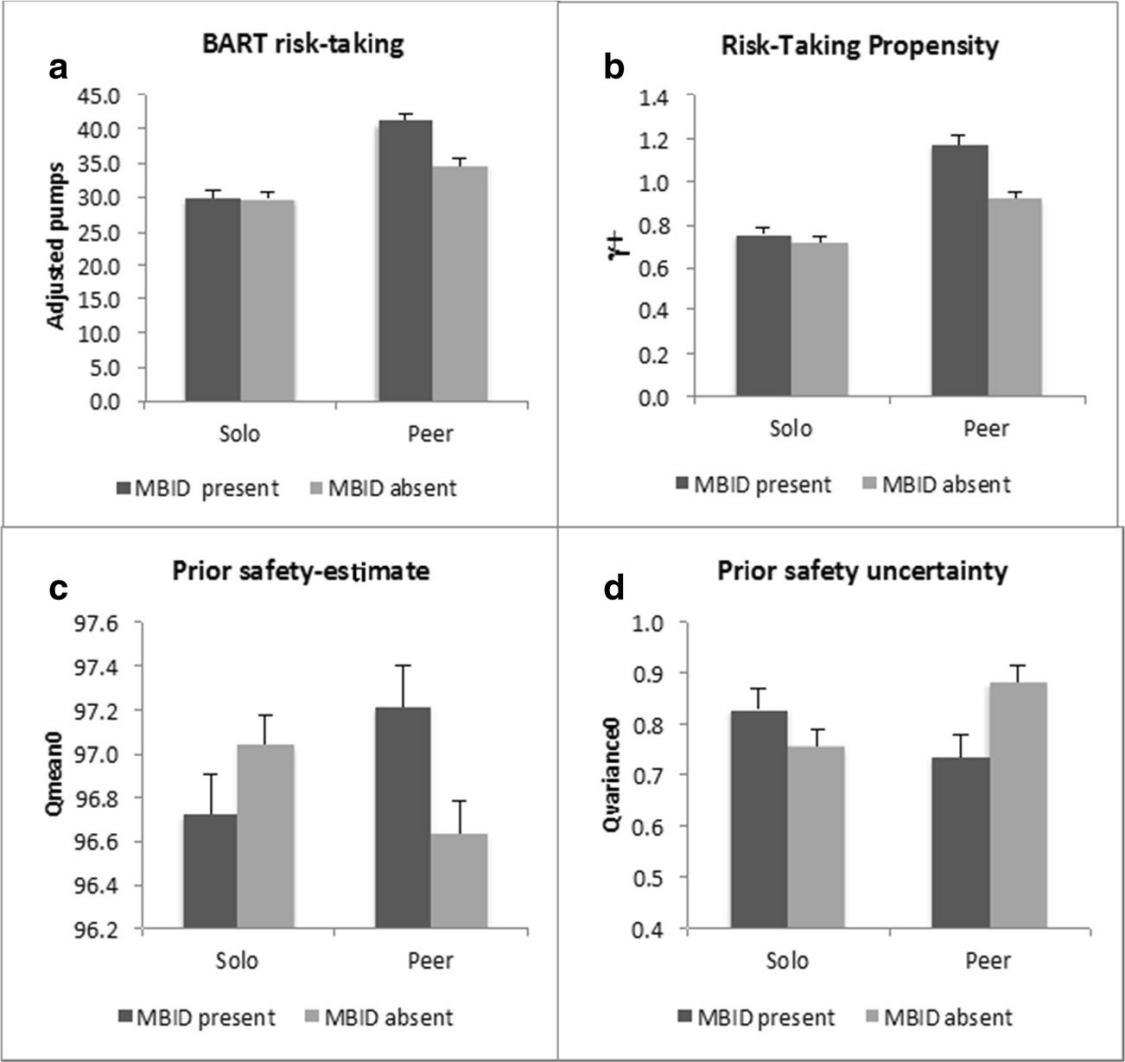
The interaction effect between Mild-to-Borderline Intellectual Disability (MBID) and BART condition on mean adjusted pumps (**a**), γ or Risk-taking propensity (**b**), Q_mean_ (**c**), and Q_variance_ (**d**). Error bars denote +1 se of the mean

The only two-way interaction that was significant was the interaction between MBID and BART condition, *F*(1, 311) = 8.03, *p* = 0.005, *partial η*^*2*^ = 0.02, that is plotted in Fig. 2a. Post-hoc analyses revealed that in the peer condition, the presence as compared to absence of MBID was associated with increased risk-taking, *F*(1, 156) = 16.65, *p*^*B*^ < 0.001*, partial η*^*2*^ = 0.10 (mean adjusted pumps 41.00 vs. 34.54, respectively). Importantly, in the solo condition, there was no such effect, *F*(1, 155) = 0.007, *p*^*B*^ > 0.99, *partial η*^*2*^ < 0.001. The remaining two-way interactions were not significant (ps > 15), and neither was the tree-way interaction, *p* = 0.45.

### Model Parameters

The second set of analyses focused on the BART model parameters, that is, risk taking propensity (γ^**+**^), behavioral consistency (*β*), safety estimation (*Q*_*mean*_*)*, and uncertainty (*Q*_*variance*_*)*. Means and standard deviations are presented in Table 3.

#### Risk-Taking Propensity

Results of the analysis on risk-taking propensity paralleled the pattern obtained for adjusted pumps. That is, there were significant main effects of MBID, *F*(1,311) = 12.50, *p* < 0.001, *partial η*^*2*^ = 0.04, and BART condition, *F*(1, 311) = 60.63, *p* < 0.001, *partial η*^*2*^ = 0.16, but not BD (*p* = 0.33). The only significant two-way interaction was between MBID x BART condition, *F*(1,311) = 7.07, *p* = 0.008, partial *η*^*2*^ = 0.02, which is plotted in Fig. 2b. In the peer condition, the presence as compared to absence of MBID was associated to increased risk-taking propensity, *F*(1, 156) = 14.32, *p*^*B*^ < 0.001, *partial η*^*2*^ = 0.08 (γ^**+**^ estimates of 1.17 vs. 0.92, respectively). In the solo condition, there was no such effect, *F*(1, 155) = 0.58, *p*^*B*^ = 0.90, *partial η*^*2*^ = 0.004. The other two way interaction were not significant (ps > 0.73) and neither was the three-way interaction (*p* = 0.91). These results thus indicate that MBID, but not BD, is related to increased risk taking propensity, but only in the peer condition.

#### Behavioral Consistency

The analysis on behavioral consistency yielded significant main effects of MBID, *F*(1, 311) = 10.76, *p* = 0.001, *partial η*^*2*^ = 0.03, and BART condition, *F*(1, 311) = 10.91, *p* = 0.001, *partial η*^*2*^ = 0.03. Presence, as compared to absence, of MBID was associated to lower behavioral consistency (mean *β*‘s were 0.080 and 0.093, respectively). In addition, behavioral consistency was lower in the peer- compared to the solo condition (mean *β*‘s were 0.079 and 0.093, respectively). The main effect of BD was not significant (*p* = 0.57) and neither were the two-way interactions (*p*s > 0.34) or the three-way interaction (*p* = 0.62). These results thus indicate that MBID and peer influence result in lower behavioral consistency.

#### Safety Estimation

**Fig. 3:**
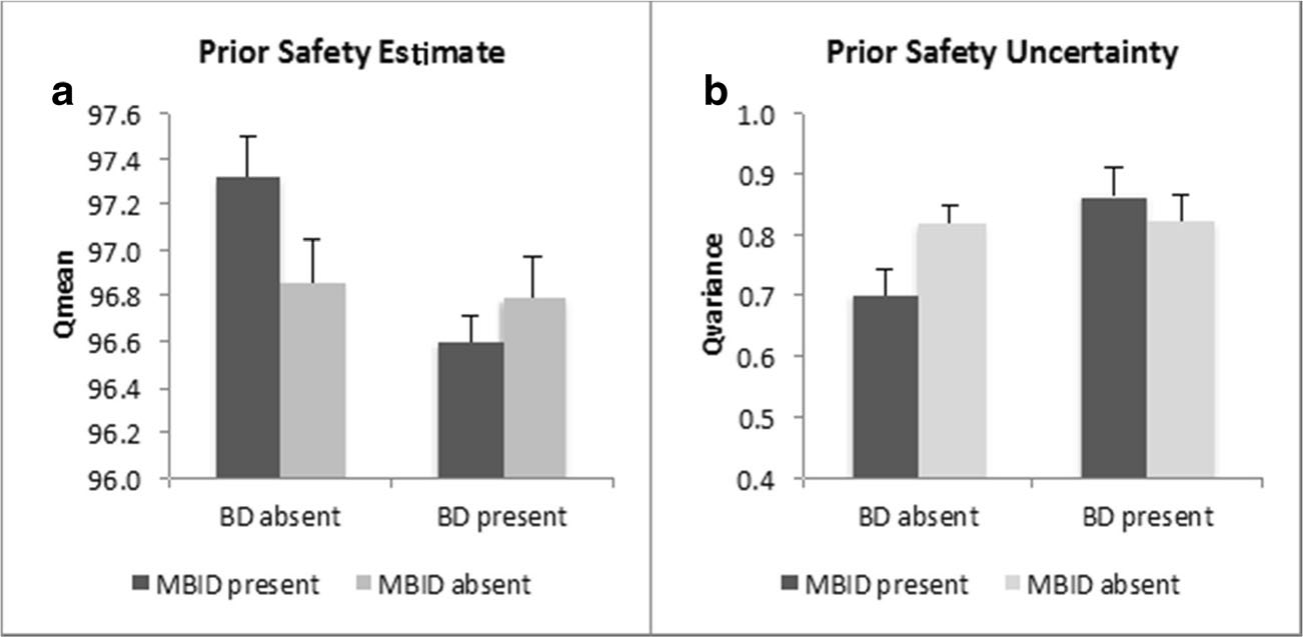
Means and standard errors for the interaction between Mild-to-Borderline Intellectual Disability (MBID) and Behavior Disorder (BD) on the prior safety estimate Q_mean0_ (**a**) and on the prior safety uncertainty Q_variance0_ (**b**). Error bars denote +1 se of the mean

The analysis on safety estimation yielded a different pattern of results (cf. Table 3). The main effect of BD was significant, *F*(1, 311) = 5.81 *p* = 0.02, *partial η*^*2*^ = 0.02, but the main effects of MBID and BART condition were not, *ps* > 0.41. The BD main effect was qualified by an interaction between BD and MBID that approached significance, *F*(1, 311) = 3.74, *p* = 0.051, *partial η*^*2*^ = 0.01, which is plotted in Fig. 3a. Post-hoc contrasts show that the increased safety estimate in the absence of BD was due to an increased safety estimate in the MBID-only as compared to the MBID+BD group (Q_mean0_’s 97.6 vs 96.4, respectively, *p*^*B*^ = 0.02). Differences between other groups were not significant (*p*^*B*^s > 0.16). The two-way interaction between MBID and BART Condition, *F*(1, 311) = 7.76, *p* = 0.006, *partial η*^*2*^ = 0.02, was significant and is plotted in Fig. 2c. Post-hoc tests confirm the pattern shown in the plot: the presence of MBID was associated to higher safety estimation in the peer condition, *F*(1, 156) = 6.15, *p*^*B*^ = 0.03, *partial η*^*2*^ = 0.04 (average Q_mean0_’s 97.2 vs. 96.6, respectively), whereas in the solo condition, there was no such effect, *F*(1, 155) = 1.94, *p*^*B*^ = 0.33, *partial η*^*2*^ = 0.01. The two-way interaction between BD and BART condition was not significant (*p* = 0.67) and neither was the three-way interaction (*p* = 0.85). Taken together these results suggest that MBID-only is related to increased safety estimates, and that MBID in general enhances safety estimates under peer pressure.

#### Uncertainty of Safety Estimation

The analysis on uncertainty showed the same pattern of results as that on safety: a main effect of BD, *F*(1, 311) = 4.71, *p* = 0.03, *partial η*^*2*^ = 0.02, but no main effects of MBID, *F*(1, 311) = 0.94, *p* = 0.33, or BART condition, *F*(1, 311) = 0.15, *p* = 0.70. The main effect of BD was qualified by an interaction between BD and MBID, *F*(1, 311) = 4.03, *p* = 0.046, *partial η*^*2*^ = 0.01, which is plotted in Fig. 3b. Post-hoc contrasts between all four MBIDxBD groups show that uncertainty was lower in the MBID-only than the MBID+BD group (Q_var0_’s 0.70 and 0.86, respectively, *p*^*B*^ = 0.03). Differences between other groups were not significant (*p*^*B*^s > 0.34). The two-way interaction between MBID and BART condition was significant, *F*(1, 311) = 7.78, *p* = 0.006, *partial η*^*2*^ = 0.02. This interaction is plotted in Fig. 2d. Post-hoc tests confirm the pattern shown in the plot: in the peer condition, presence of MBID was associated with significantly lower uncertainty, *F*(1, 156) = 6.98, *p*^*B*^ = 0*.*02, *partial η*^*2*^ = 0.04 (average Q_variance0_’s of 74 and 0.88, respectively). In the solo condition, there was no such effect, *F*(1, 155) = 1.68, *p*^*B*^ = 0.39, *partial η*^*2*^ = 0.01. The two-way interaction between BD and BART condition was not significant (*p* = 0.87) and neither was the three-way interaction (*p* = 0.84). Taken together these results suggest that MBID-only is related to decreased uncertainty estimates, and that MBID in general decreases uncertainty estimates under peer pressure.’

## Discussion and Conclusion

This study assessed whether individual differences in intellectual functioning and behavior disorder are associated to risk-taking in situations in which peer influence is absent or present. To this end, adolescents with Mild-to-Borderline Intellectual Disability (MBID) and/or Behavior Disorder (BD) were compared to typical controls on the Balloon Analogue Risk Task in either a solo or a peer condition. The primary finding of the study was that MBID was related to increased risk taking, but only under peer-influence, whereas BD was not related to increased risk-taking in either a solo or the peer context. These results suggest that MBID related increased real-life risk-taking such as delinquency and sexual-risk-taking, may be better explained by low intellectual functioning than by comorbid BD. In addition, these results suggest that this increased real-life risk taking may not originate in increased risk taking per se, but may rather be related to risk-taking under peer-influence, which is a complex, multifaceted risk-taking context (Erickson and Jensen 1977; Gardner and Steinberg 2005). It has been argued before that individuals with intellectual disability are especially credulous (Greenspan et al. 2011) which may be related to delays in social cognition (Abbeduto et al. 2004; Collot d’Escury 2007). Together with low feelings of self-efficacy (Khemka and Hickson 2006), this may increase reliance on others for making decisions about whether to partake in risk-taking behavior (Greenspan et al. 2011). This could be a potential explanation for increased susceptibility to peer-influence in MBID that should be further studied by including measures of social-cognition in studies of peer-influence on risk-taking.

However, there is another potential explanation for why risk-taking under peer-influence may have been increased in MBID. In the peer-condition additional stimuli (i.e., audio and speech bubbles) were introduced, that were absent in the solo condition. As adolescents with MBID have more difficulty with suppressing irrelevant task information and have a more limited working memory (Bexkens et al. 2014a; Van der Molen et al. 2010), this could have interfered with their performance more than in adolescents without MBID. Distracting information could potentially interfere with the ability to weigh-up all the decision information which may lead to more risky decision-making. Further research will indicate whether this is a possible confound of our paradigm, or actually increases the ecological validity of the task since this is similar to real life where the context is seldom unidimensional. The possibility that distracting information may increase risk-taking has also been posed by Peake et al. (2013). They argued that adolescents with low resistance to peer-influence may be distracted by the social implications of their decisions, and may therefore take more risky decisions.

A special asset of this study was the use of formal modelling allowing a more fine-grained analysis of the MBID related increase in risk-taking under peer-influence. Specifically, this analysis showed that MBID increased the effects of peer-influence on the key parameter, risk-taking propensity, a parameter which has been associated to a variety of risk taking behaviors (Cavanagh et al. 2012; Rolison et al. 2012; Wallsten et al. 2005). In addition to that, the MBID-only group was characterized by increased safety estimates and decreased uncertainty in these estimates if peer influence was present. These results thus indicate that the MBID-only group is characterized by an additional deficit when under peer influence: in addition to an enhanced risk taking propensity, they are characterized by increased safety estimates, which also lead to increased risk taking behavior.

The fact that increased safety perception and certainty seemed specific to the MBID-only and not the MBID+BD group is interesting, as these groups did not differ on IQ-scores. It has been argued before that adolescents with MBID+BD may function higher intellectually than adolescents with MBID-only (Bexkens et al. 2014b; Ponsioen and Van der Molen 2002) - that is, tests of intellectual functioning may underestimate IQ in this group because behavioral difficulties interfere with intellectual testing results. Although speculative, it would explain larger deficits in adolescents with MBID-only, which were not only found in the present study, but also in studies examining cognitive decision-making (Bexkens et al. 2016), inhibitory control (Bexkens et al. 2014b; Ponsioen and Van der Molen 2002), and social cognition (Ponsioen and Van der Molen 2002; Proctor and Beail 2007). In this regard, there is a strong need for replication and in-depth comparison of these two groups.

In contrast to expectations, adolescents with BD did not exhibit increased risk-taking or increased susceptibility to peers. These findings are inconsistent with studies that do show increased risk-taking in BD on experimental risky decision tasks (Dekkers et al. 2016; Humphreys and Lee 2011; Schutter et al. 2011), but are consistent with other studies that also do not find these effects (Marini and Stickle 2010; Swogger et al. 2010; Weafer et al. 2011). A potential explanation may be found in how participants perceived the task in terms of reward, as it has been argued that increased risk-taking in BD may be particularly associated with increased reward sensitivity (Alegria et al. 2016; Von Rhein et al. 2015). In the present study, participants were only rewarded upon completion of the task. As adolescents with BD tend to show steep discounting of future compared to immediate rewards, this may have influence the reward value of the task for adolescents with BD (White et al. 2014).

Another possible explanation for the lack of effect in the BD group may be related to medication use or participation in other non-pharmacological interventions. Although we had no data on the latter, it is possible that adolescents in BD groups may have participated in these types of interventions to reduce (impulsive) problem behavior. Although this may have potentially reduced differences in solo risk-taking between the groups, it notably did not fully reduce susceptibility to peer-influence in the MBID+BD group, as there was still an effect of peer condition in that group.

Note that generalizability of these results is limited to male adolescents as no female adolescents were included in the present study. Recent findings on gender differences in risk-taking indicate that gender differences (i.e., increased risk-taking in males) may mainly exist on real-life measures, and less so on laboratory measures of risk-taking (Duell et al. 2017). However, there is also some evidence that seem to suggest that males and females approach experimental gambling tasks differently (Van Hoorn et al. 2016a). Future studies on risk-taking in MBID should include both male and female adolescents.

A limitation of this study is that we used only one type of risk-taking task and that we did not relate BART performance to real life risk-taking. Although BART performance was previously found to be associated with real-life risk-taking (Hunt et al. 2005; Lejuez et al. 2007, 2002; MacPherson et al. 2010; Mishra et al. 2010; Wallsten et al. 2005) it would be a good addition if future MBID studies included measures of real-life risk tasking as well. As adolescents with MBID can experience difficulty with self-report (Emerson et al. 2013), the focus in such studies should be on other informants such as parents or teachers or on more objective measures of real-life risk-taking such as records of substance abuse or delinquency.

Similar to other experimental studies of peer-influence on risk-taking, the present paradigm provides no information about the specificity of the peer-effect in adolescents with MBID. Further research is needed for instance, to pinpoint whether the effects are peer-specific, or whether adult influencers would have the same effect. Previous research in typically developing adolescents showed that early adolescents may be particularly susceptible to the opinions of peers compared to opinions of adults (Knoll et al. 2015). In addition, an important question would be whether peer context can also positively influence behavior of adolescents with MBID. The potential positive effect of prosocial peer-influence has already been demonstrated in experimental study in typically developing adolescents and adolescents with autism (Van Hoorn et al. 2016b, 2017).

The current results point to interventions that may reduce risk taking in adolescents with MBID. Interventions that would for example increase resistance to peer-influence by training neurocognitive skills, such as cognitive control (cf. Van der Molen et al. 2010) may be effective. An alternative intervention strategy may be to use enhanced susceptibility to peer-influence to reduce risk taking. More specifically, interventions may focus on strengthening the social context of adolescents with MBID with risk-discouraging peers. It has been established in typical developing populations that pro-social peers can successfully reduce externalizing behavior (Witvliet et al. 2009). Efficacy of these interventions in reducing risk-taking in adolescents with MBID will need to be investigated.

In conclusion, the present laboratory study suggests that 1) MBID related increased real-life risk-taking may be better explained by low intellectual functioning than by comorbid BD, and 2) may not originate in increased risk taking per se, but rather in increased risk-taking propensity and estimation of safety under peer-influence, which is a complex, multifaceted risk-taking context. Although the found effects were modest, and should be confirmed in further research, even a small effect may have large implications in this vulnerable population. Reduction of susceptibility to peer-influence may therefore act as a potentially powerful target in reducing risk taking is adolescents with MBID.

## Footnotes

1 Although a Rhat value of 1.0 is commonly used as a cut-off to determine preciseness of fit, we chose to use a cut-off of 1.5 for two reasons. First, as we tested clinical groups, we expected the data to be noisier and therefore the Rhat values to be a bit higher than when testing typically developing samples. Second, we chose a cut-off of 1.5, because this provided a good compromise between quality of the fits and statistical power for the analysis. Including participants with Rhat values larger than 1.5 did not change the pattern of results, but did decrease power of the analysis.

2 To check whether the peer-manipulation affected risk-taking in all groups, we performed post-hoc tests on the effect of BART condition in each group. These indicated that the main effect of BART condition was present in all groups (*p*^B^s < 0.03) except for the BD-only group (*p*^B^ = 0.29).
